# Conjugation of Functionalized Gold Nanorods and Copper (I)-Based Drug: An Anisotropic Nano Drug Delivery System

**DOI:** 10.3390/nano16030217

**Published:** 2026-02-06

**Authors:** Elena Olivieri, Simone Amatori, Chiara Battocchio, Giovanna Iucci, Martina Marsotto, Diego Lipani, Annarica Calcabrini, Marisa Colone, Annarita Stringaro, Maria Luisa Dupuis, Giuseppe Ammirati, Alessandra Paladini, Francesco Toschi, Maura Pellei, Carlo Santini, Miriam Caviglia, Jo’ Del Gobbo, Luca Tortora, Eleonora Marconi, Valentin-Adrian Maraloiu, Iole Venditti

**Affiliations:** 1 Sciences Department, Roma Tre University, Via della Vasca Navale 79, 00146 Rome, Italy; elena.olivieri@uniroma3.it (E.O.); simone.amatori@ceric-eric.eu (S.A.); chiara.battocchio@uniroma3.it (C.B.); giovanna.iucci@uniroma3.it (G.I.); martina.marsotto@uniroma3.it (M.M.); diego.lipani@unirom3.it (D.L.); luca.tortora@uniroma3.it (L.T.); eleonora.marconi@uniroma3.it (E.M.); 2 CERIC-ERIC, S. S. 14-km 163.5 in AREA Science Park, 34149 Basovizza, Italy; 3 Department of Chemical Science and Technologies, Tor Vergata University of Rome, Via della Ricerca Scientifica 1, 00133 Rome, Italy; 4 National Center for Drug Research and Evaluation, Istituto Superiore di Sanità, 00161 Rome, Italy; annarica.calcabrini@iss.it (A.C.); marisa.colone@iss.it (M.C.); annarita.stringaro@iss.it (A.S.); marialuisa.dupuis@iss.it (M.L.D.); 5 CNR—Istituto di Struttura della Materia (CNR-ISM), EuroFEL Support Laboratory (EFSL), 00100 Rome, Italy; giuseppe.ammirati@cnr.it (G.A.); alessandra.paladini@cnr.it (A.P.); francesco.toschi@cnr.it (F.T.); 6 School of Science and Technology, University of Camerino, Via Madonna delle Carceri (ChIP), 62032 Camerino, Italy; maura.pellei@unicam.it (M.P.); carlo.santini@unicam.it (C.S.); miriam.caviglia@unicam.it (M.C.); jo.delgobbo@unicam.it (J.D.G.); 7 National Institute of Materials Physics, 405A Atomistilor St., 077125 Bucharest, Romania; maraloiu@infim.ro1

**Keywords:** gold nanorods, copper(I)-based drug, drug delivery system, MTT assay, DLS, SR-XPS, NEXAFS

## Abstract

Gold nanorods (AuNRs) were synthesized and optimized with the aim of obtaining strongly hydrophilic nanomaterials, suitable as a drug delivery system (DDS) for copper-based drugs. After careful purification, AuNRs were characterized by ultraviolet–visible–near-infrared spectroscopy (UV–Vis–NIR), showing two typical localized surface plasmon resonance (LSPR) bands in the range 550–750 nm. Fourier Transform Infrared (FT-IR) and high-resolution X-ray photoelectron (HR-XPS) spectroscopies verified the surface functionalization. Transmission electron microscopy (TEM) showed AuNRs with regular shape and size, with an aspect ratio (AR) of 2.6. Dynamic Light Scattering (DLS) measurements confirmed the size and the stability in water for up to 3 months. The AuNRs were conjugated with copper(I) drugs, i.e., [Cu(PTA)_4_]BF_4_ (PTA = 1,3,5-triaza-7-phosphadamantane). The drug loading procedures and efficiency were optimized, and the best loading was η (%) = 50 ± 7%. The non-covalent interactions of the Cu(I) complex with the AuNRs were studied by means of UV–Vis–NIR, ζ-potential, HR-TEM, FT-IR, synchrotron radiation-induced X-ray photoelectron (SR-XPS), and near-edge X-ray absorption fine structure (NEXAFS) spectroscopy measurements. The MTT assay performed on Vero E6 cells showed that AuNRs and AuNR-Cu(I) conjugates had no significant effect on cell viability, being biocompatible, causing a reduction in cell viability only after prolonged exposure.

## 1. Introduction

In the last ten years, nanomaterials have been employed with great success in several areas, including catalysis optics, energy, sensors, and biotechnology [1,2,3,4,5,6,7]. In this latter field, gold nanoparticles in particular are enjoying great success, thanks to their simple and versatile synthesis and their high biocompatibility. Among others, gold nanorods (AuNRs) are being studied in biomedical applications, including photothermal therapy, gene/drug transport, and imaging [8,9,10,11,12,13,14,15]. This success is, in part, thanks to the ease with which it is possible to manipulate the AuNR dimensions and aspect ratios (ARs). In addition, their surface can be modified, allowing for conjugation to biomolecules for specific targeting and absorption and/or delivery of drugs [16,17,18,19]. To use them for biomedical purposes, it is very important to understand how the different physical and chemical properties of AuNRs have an impact on cells, especially with respect to the presence of surface stabilizers that strongly influence any toxicity and absorption. A particular feature of AuNRs is a double localized surface plasmon resonance (LSPR), one resulting from the excitation of the oscillating electrons transverse to the main axis of the rod and the other from electrons oscillating in the longitudinal direction. The two plasmons have peaks centered around 520 nm and in the near infrared area, from 680 to 1200 nm. By varying the AR, a variation in the position of plasmons is obtained, where the larger the AR becomes, the larger the λ_max_ of the LSPR of the second peak. The advantage of using longer AuNRs for diagnostic–therapeutic purposes depends on the possibility of exciting and tracing them in a simple way through NIR spectroscopy, in which the energy range is close to the “biological window”, where the absorption of light radiation by water and hemoglobin, and therefore, by the tissues of the human body, is negligible: this opens up a range of possibilities for the use of AuNRs in the biomedical field [20,21,22,23,24].

The recent literature has shown promising antimicrobial potential for copper(I) and copper(II) complexes, where ligand design, coordination geometry, and metal–ligand interactions also influence biological efficacy [25,26]. These complexes have demonstrated significant antibacterial and antifungal activity, often superior to or equal to standard drugs in biological studies. The integration of nanotechnology-based delivery and delivery systems could improve bioavailability and target specificity, opening new avenues in the treatment of drug-resistant infections.

The immortalized cell line (Vero) established from kidney epithelial cells of the African green monkey (*Chlorocebus sabaeus*) was used in our study for biological tests. The Vero cell line represents one of the most common mammalian immortalized cell lines used as a model in many research applications (high-throughput screenings, 3D cell cultures, drugs/chemicals testing, bioproduction) [27,28]. Moreover, Vero cells, along with their various derivatives, are the most widely used cell culture for the replication of viruses such as SARS-CoV-2, antiviral screening, and virological studies. This is because they offer many advantages: they are easy to maintain, offer high levels of infectivity, and support efficient virus replication and production [29,30]. There are several commercially available Vero cell lines (e.g., Vero, Vero 76, and Vero E6), all derived from the same source. In particular, in our study, the subline E6 (Vero E6) was employed to evaluate the effects of AuNRs and AuNR-Cu(I) conjugates on cell viability to assess their biocompatibility in view of antiviral applications [31].

In this framework, the synthesis of AuNRs was presented with the aim of obtaining strongly hydrophilic anisotropic nanomaterials, suitable for drug delivery and photothermal therapy. AuNRs were synthesized by seed-mediated methods in two steps, and, after careful purification, they were investigated by means of UV–Vis–NIR, FT-IR and synchrotron radiation-induced X-ray photoelectron (SR-XPS) spectroscopies. High-resolution transmission electron microscopy (HR-TEM) observations confirmed nanosizes in a range useful for our biomedical applications. Moreover, conjugated systems with copper-based drugs, i.e., [Cu(PTA)_4_]BF_4_, were prepared. The drug loading procedures and efficiency (η) were optimized to improve the hydrophilicity and bioavailability of DDS. The best optimized loading is η (%) = 50 ± 10%. The non-covalent interactions of the Cu(I) complex with the AuNR surface were studied using UV–Vis–NIR, FESEM-EDX, FT-IR, SR-XPS and NEXAFS measurements. Moreover, the MTT tests performed with Vero cells allowed us to study their biocompatibility in view of antiviral applications.

## 2. Materials and Methods

### 2.1. Materials

**Figure 1:**
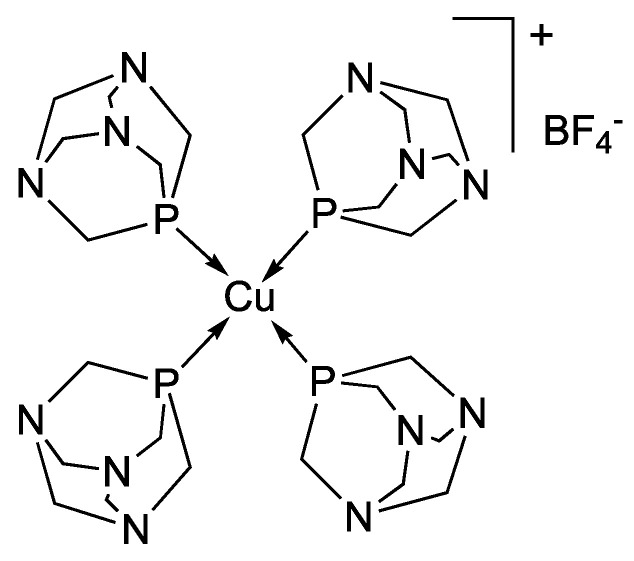
Chemical structure of the copper(I) complex [Cu(PTA)_4_]BF_4_.

Cetyltrimethylammonium bromide (C_19_H_42_BrN, CTAB, ≥97% Millipore Corporation Merck Italy), tetrachloroauric(III) acid trihydrate (HAuCl_4_·3H_2_O, ≥99.9%, Sigma-Aldrich, Italy), sodium borohydride (NaBH_4_, 99.99%, Sigma-Aldrich), silver nitrate (AgNO_3_, 99.9%, Carlo Erba, Italy) and L-ascorbic acid (C_6_H_8_O_6_, 99%, Sigma-Aldrich) were used as received. Bidistilled water was used in all the procedures. All the other products were reagent-grade chemicals and were used as received by Merck Italy, without further purification. The analytically pure copper(I) complex [Cu(PTA)_4_]BF_4_ (Figure 1) was synthesized by a one-step reaction of [Cu(CH_3_CN)_4_]BF_4_ with an excess of PTA in an acetonitrile solution at room temperature. The reaction mixture was stirred overnight and filtered, and the white residue was washed with chloroform and diethyl ether and then dried under vacuum [32,33].

### 2.2. AuNR Synthesis

CTAB-stabilized AuNRs were prepared with a reliable procedure in two steps based on the literature [34,35]. First step: HAuCl_4_ 3H_2_O (5 mL, 5 × 10^−4^ M) and CTAB (5 mL, 0.2 M) water solutions were mixed and degassed by Ar for 5 min. Then, NaBH_4_ water solution (0.6 mL, 10^−2^ M) was added as a reducing agent to obtain the seed solution in 20 min. Second step: CTAB solution (5 mL, 0.2 M), HAuCl_4_∙3H_2_O solution (5 mL, 10^−3^ M), and AgNO_3_ solution (0.2 mL, 4 × 10^−3^ M) were mixed (molar ratio Au/CTAB/Ag = 1/200/0.16) under vigorous stirring and with an inert atmosphere (Ar). After 5 min, ascorbic acid solution (0.07 mL, 0.078 M) was added (molar ratio Au/Asc.Ac. = 1/1.092), followed by 0.024 mL of a seed solution. Gradually, the solution turns into a reddish-violet color. The product is left at room temperature (25° C) for 24 h and then purified by centrifugation (13,000 rpm, 15 min, twice washing with deionized water). For more experimental details, see Supporting Information Table S1. Main characterizations: UV–Vis (λ_max_ (nm), H_2_O) T-SPR, 515 nm; L-SPR, 740 nm; ζ potential: 46 ± 4 mV; TEM dimensions: transversal side, 20 ± 5 nm, longitudinal side, 60 ± 15 nm.

### 2.3. AuNR-Cu(I) Conjugate Preparation

[Cu(PTA)_4_]BF_4_ was prepared according to procedures reported in the literature [32,36]. The AuNRs and the Cu(I) complex were mixed in water (Au/Cu = 6/1 *w*/*w*) under gentle stirring (room temperature, 24 h), and then, the suspension was centrifuged (13,000 rpm, 15 min, twice washing with water) to obtain AuNR-Cu(I) as a solid residue. It was stored at T = −18 °C, while the supernatant was used for loading evaluation. Loading efficiencies (η) were calculated by using calibration curves, as in our previous works [37,38]. For each sample, at least 3 independent measurements were carried out, and the mean value and standard deviation were reported. For more experimental details, see Table S2.

### 2.4. Characterizations

UV–Vis spectra were acquired with a Shimadzu 2401 PC spectrophotometer (200–800 nm), while NIR spectra were obtained with a Nicolet IS50 FT-IR Thermo Scientific, Waltham, MA, USA, spectrophotometer (400–1100 nm) using quartz cells with 1 cm of optical path. FT-IR spectra were acquired in transmittance mode (4000–400 cm^−1^) with a Bruker Vector 22 using pellets made of the sample and KBr. The ζ potential distribution of AuNRs in H_2_O was investigated by means of Zetasizer Ultra Red, Malvern, UK [39,40,41,42,43]. For TEM analysis, images were obtained with a Philips EM 208S instrument (FEI-Thermo Fisher, Waltham, MA, USA, operating at 100 kV, equipped with a Mega-view II SIS Olympus camera; others, with a probe-corrected JEOL JEM ARM200F microscope, Peabody, MA, USA, operated at 200 kV, equipped with a Gatan, UK, Ultrascan CCD camera. Samples were drop-casted on formvar/carbon-supported copper grids, and after a few minutes, the excess liquid was blotted with filter paper, and then the samples were examined.

Synchrotron radiation-induced X-ray photoelectron spectroscopy (SR-XPS) measurements were performed at the SuperESCA beamline at the ELETTRA facility in Trieste (Italy). Photoemission data were collected in fixed analyzer transmission mode (pass energy = 50 eV), with the monochromator entrance and exit slits optimized at 30 and 20 mm, respectively. A photon energy (PE) of 360 eV was used for C1s, P2 and Au4f spectra; for N1s and Ag3d spectral regions, a PE of 520 eV was selected; finally, Cu2p spectra were acquired using a PE of 1100 eV to maximize signal intensities. The total binding energy resolution was about 0.22 eV. As a reference, the energy scale was calibrated using the C1s aliphatic signal at 285.00 eV and the Au4f_7/2_ signal of AuNRs arising from metallic gold atoms, always found at 83.96 Ev [44]. Curve-fitting analysis of the C1s, N1s, P2p, Au4f, Ag3d and Cu2p spectra was performed using Gaussian curves as fitting functions. All spin–orbit doublets were fitted by using the same full width at half-maximum (FWHM) for each pair of components of the same core level. A spin–orbit splitting of 6.0 eV and a branching ratio of Ag3d_5/2_/Ag3d_3/2_ = 3/2 were selected for Ag3d doublets; a spin–orbit splitting of 3.7 eV and a Au4f_7/2_/Au4f_5/2_ = 4/3 branching ratio were used for Au4f components; branching ratios of P2p,Cu2p_3/2_/_1/2_ = 2/1 and spin–orbit splittings of 0.8 eV and 19.8 eV were chosen for, respectively, P2p and Cu2p doublets. When several different species were identified in a spectrum, the same FWHM value was used for all individual photoemission bands. To perform SR-XPS analysis, pristine Cu(I) complex ([Cu(PTA)_4_]BF_4_), AuNRs and conjugates (AuNR-Cu(I)) were deposited onto TiO_2_/Si(111) wafer substrates with a drop-casting procedure.

Near-edge X-ray absorption fine structure (NEXAFS) measurements were performed at the BEAR beamline (Bending magnet for Emission Absorption and Reflectivity) at the ELETTRA storage ring, installed at the left exit of the 8.1 bending magnet exit. The apparatus is based on a bending magnet as a source and beamline optics delivering photons from 5 eV up to about 1600 eV with a selectable degree of ellipticity. In these experiments, we used ammeters to measure the drain current from the sample. C and N K-edge spectra were collected at magic (54.7°) incidence angles of the linearly polarized photon beam with respect to the sample surface. The photon energy and resolution were calibrated and experimentally tested at the K absorption edges of Ar, N2 and Ne. The raw C and N K-edge NEXAFS spectra were normalized to the incident photon flux by dividing the sample spectrum by the spectrum collected on a freshly sputtered gold surface. Spectra were then normalized, subtracting a straight line that fits the part of the spectrum below the edge and assigning the values at 330.00 and 420.00 eV to 1 for C and N, respectively.

### 2.5. Cell Cultures and Biological Tests

For cell viability assessment, the Vero E6 cell line was employed. This cell line, from the American Type Culture Collection (ATCC), was kindly provided by Dr. Andrea Cara (National Center for Global Health, Istituto Superiore di Sanità, Rome, Italy). Vero cells are normal kidney epithelial cells isolated in 1962 from an African green monkey (*Chlorocebus sabaeus*). They did not originate from a tumor, but they became a continuous/immortalized cell line through spontaneous mutations during culture. Vero cells are characterized by an aneuploid karyotype (abnormal chromosome number), typical of immortalized lines. Cells were grown in DMEM (Dulbecco’s Modified Eagle’s Medium, Euroclone, Thermo Fisher Scientific, Waltham, MA, USA) supplemented with 10% fetal bovine serum (FBS) (Corning, Charlotte, NC, USA), 1% penicillin (50 U-mL)–streptomycin (Gibco), 1% non-essential amino acids (Euroclone), 1% Sodium Pyruvate (Gibco) at 37 °C, and in an atmosphere containing 5% CO_2_ and sub-cultivated at confluence. To evaluate the effects of AuNRs and AuNR-Cu(I) conjugate treatments on Vero E6 cell viability, the MTT assay was performed. Cells were seeded into a 96-well plate (Nunclon, Thermo Fisher Scientific, Waltham, MA, USA) at a density of 1.8 × 10^4^ cells/well. After 24 h, cells were exposed to different concentrations of AuNRs and AuNR-Cu(I) conjugates (0.025, 0.05, 0.25, 0.5 and 1 µg/mL) in cell culture medium for 24, 48 and 72 h. Then, the medium was removed. and 100 µL of MTT (3-(4,5-dimethylthiazol-2-yl)-2,5-diphenyltetrazolium bromide) solution (Sigma-Aldrich, USA) (0.5 mg/mL) was added to each well for 2 h. At the end of this period, cells were dissolved by adding 100 µL/well of DMSO (Merck, Germany). Absorbance was read at 570 nm by a microplate reader (Varioskan™ LUX multimode, Thermo Fisher Scientific, Waltham, MA, USA). Data were expressed as a relative % of cell viability, calculated as the ratio of the absorbance value of the treated cells to the absorbance value of the control cells [(absorbance_treated_/absorbance_control_) × 100]. All experiments were performed in triplicate in at least three independent experiments.

### 2.6. Statistical Analysis

In cell viability experiments, the results are expressed as mean ± SD. Statistical data analysis was performed using the GraphPad PRISM 10 software (unpaired *t*-test). Statistical significance was assessed by the one-way ANOVA test, followed by the Bonferroni test or the Tukey test. The differences in the means are considered statistically significant with *p*-values ≤ 0.05.

## 3. Results and Discussion

### 3.1. AuNR Synthesis

**Figure 2:**
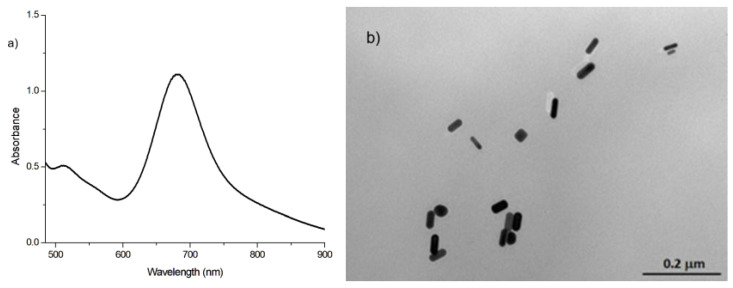

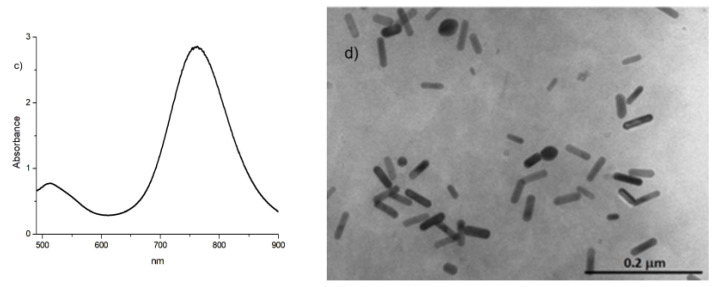
(**a**) NIR of AuNRs-1 with λ_max_ 515 nm and 683 nm; (**b**) TEM image of AuNRs-1 with A.R. = 2.5; (**c**) NIR of AuNRs-2 with λ_max_ 518 nm and 778 nm; (**d**) TEM image of AuNRs-2 with A.R. = 3.8.

As is well known, in AuNR synthesis, the size and aspect ratio of the particles can be modulated by varying the experimental parameters, such as the temperature, time, capping agent, reducing agent, and molar ratios of the various reagents used [34,35]. Moreover, suitable surface functionalization can achieve high hydrophilicity, which is the main aim for drug delivery applications. In such cases, AuNRs can act as a vehicle to achieve a targeted and controlled release of the drug, in many cases also improving bioavailability [45,46,47,48]. The synthesis proposed here to obtain strongly hydrophilic AuNRs is based on the use of a seed and involves two steps, both in the aqueous phase. In the first step, the seed is produced starting with Au^3+^ ions, using a strong reducing agent, such as sodium boron hydride, and a capping agent, such as CTAB. In this step, small-sized particles are produced, which will be used in the second step. Specifically, in the following step, always in the presence of CATAB and Au^3+^ ions, a milder reducing agent, ascorbic acid, silver ions, and the seed were added to the mix. These conditions promote the growth of anisotropic particles, with peculiar optical properties, such as two surface plasmon resonance peaks (two LSPR bands) related to the length/thickness ratio or aspect ratio (AR): the transverse and longitudinal bands (T-LSPR and L-LSPR). AuNRs are more easily polarized longitudinally, meaning that LSPR occurs at a lower energy and, thus, a higher wavelength. As the aspect ratio (ratio of length to width) of a nanorod is increased for a fixed diameter, the L-LSPR and T-LSPR are both affected; however, the longitudinal axis is more polarizable and more sensitive to aspect ratio changes. In AuNRs, the L-LSPR wavelength can be tuned from 550 nm to over 2000 nm by adjusting to longer aspect ratios, while the T-LSPR remains relatively constant at ~510–520 nm. It is reported in the literature that AuNRs can show a shift of a few nanometers in the T-LSPR absorption band (505–520 nm), induced by an increase in the aspect ratio from 1.7 to 5.2 [49,50]. On the other hand, the L-LSPR can move from 590 to 935 nm when the aspect ratio increases from 1.7 to 5.2. The critical experimental parameters to control the aspect ratio are mainly in the second step and are the amount of reducing agent (i.e., ascorbic acid), the amount of AgNO_3_, and the volume of the seed solution used. Regarding ascorbic acid (AA), it is possible to note an increase in the length of the nanorods when the concentration of the reducing agent decreases. In fact, by using a greater quantity of AA, the speed at which Au^3+^ is reduced is faster, and the growth rate of the AuNRs is faster; therefore, shorter AuNRs are formed. Figure 2 shows two different sizes of AuNRs obtained by using different AA volumes (0.078 M) during the second step of the synthesis: for AuNRs-1, the AA volume = 140 µL, and for AuNRs-2, the AA volume = 175 µL. As expected, the dimensions increase by decreasing the reducing agent amount, i.e., AA, the distance between the transverse and longitudinal plasmon peaks increases from 168 to 260 nm, and the AR increases from 2.5 for AuNR-1 to 3.8 for AuNRs-2.

**Figure 3:**
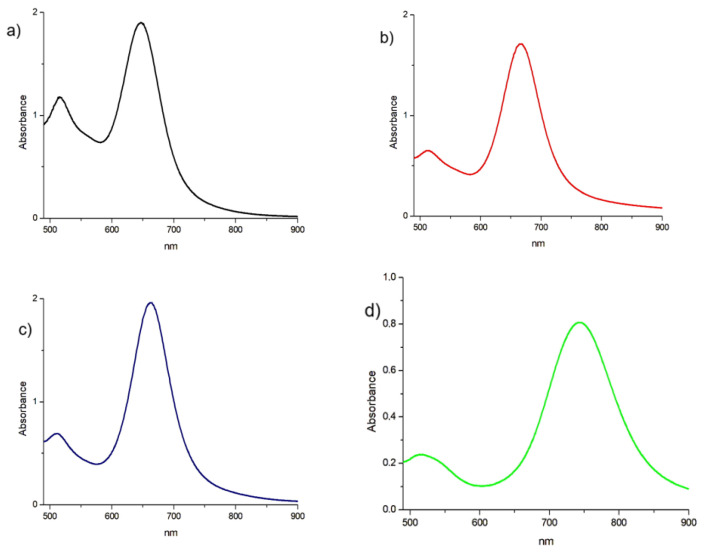
Vis–NIR spectra of AuNRs using different experimental conditions in the second step: (**a**) AuNRs with λ_max_ 520 and 654 nm using [AgNO_3_] = 0.002 M; (**b**) AuNRs with λ_max_ 520 and 670 nm using [AgNO_3_] = 0.008 M; (**c**) AuNRs with λ_max_ 516 and 662 nm using volume of seed solution = 12 µL; (**d**) AuNRs with λ_max_ 518 and 744 nm using volume of seed solution = 24 µL.

The main parameter that must be modulated in order to obtain nanorods of a desired size is the amount of AgNO_3_. The role of AgNO_3_ is linked to the anisotropic growth of the rods since, as explained in the literature, the silver is incorporated into the mass of the nanorods by binding the Br of the CTAB. To study the effect of this parameter, different concentrations of AgNO_3_ were used, and it was observed that as the concentrations increased, two plasmons at distant wavelengths were highlighted, as also reported in the literature. However, the addition of AgNO_3_ above a certain threshold limits the growth of the rod [51,52]. The volume of seed solution used also influenced the growth of the AuNRs, in a proportional way: smaller nanorods correspond to lower concentrations of seed solution. Figure 3 shows this typical trend of the UV–Vis–NIR spectra as the quantities of AgNO_3_ and seed solution vary, which also confirms the proposed synthetic protocol that was expected from the literature [53,54].

### 3.2. Cu(I)-Complex Loading on AuNRs

**Figure 4:**
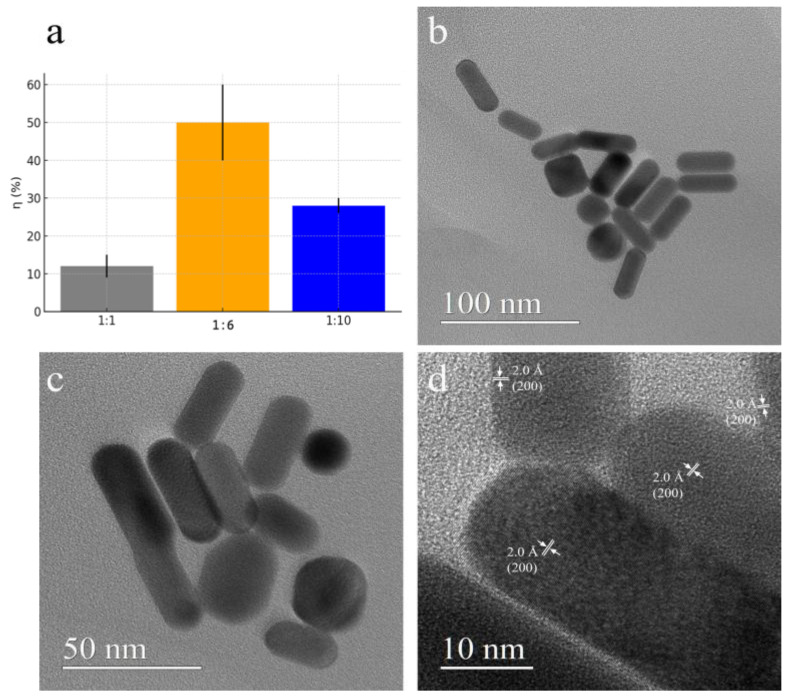
Conjugated system: (**a**) loading percentage of Cu(I) complex on AuNRs by putting different amounts (weight/weight ratio) in contact for 24 h: in gray, a 1:1 ratio with η (%) = 12 ± 3%; in orange, 1:6 with η (%) = 50 ± 7%; and in blue, 1:10 with η (%) = 28 ± 2%; (**b**,**c**) CTEM images show that the predominant morphology is nanorods; (**d**) HRTEM image of well-crystallized AuNRs.

The synthesized AuNRs were found to be highly hydrophilic and stable in an aqueous environment, as confirmed by UV–Vis and ζ potential measurements performed immediately after synthesis and after 3 months (see Supporting Information, Figure S1). These AuNRs are particularly suitable for drug delivery, and in this study, the aim is to conjugate them with the Cu(I) complex [Cu(PTA)_4_]BF_4_ to increase the final bioavailability. The loading protocol was based on the simple physical contact of AuNRs and Cu(I) complexes that can be physically adsorbed [37,46]. On the basis of these considerations, the loading protocol for AuNRs and the Cu(I) complexes was performed in a water solution at room temperature (25 °C) under gentle stirring. In Figure 4a, the value of the loading efficiency, η (%), was reported for different Cu(I)/Au *w*/*w* ratios: the best result was obtained from the ratio Cu(I)/Au = 1/6, reaching an η (%) of 50 ± 7%. The conjugate is prepared with a gold/copper *w*/*w* ratio of Au/Cu(I) = 6/1, and since the loading efficiency is 50%, the conjugate will have a *w*/*w* ratio of Au/Cu(I) = 12/1. Therefore, for every mg of AuNR-Cu(I) conjugate, about 0.08 mg is copper. The colloidal stability was confirmed by Z potential measurements after 9 months (data reported in SM-Figure S1b).

Morpho-structural investigations were performed on the conjugate with the highest loading, and some conventional TEM (CTEM) images are reported in Figure 4b–d, showing regular shape particles and low aggregation phenomena. These images show nanorods with a diameter ranging from 14 to 19 nm and a length from 32 nm to 43 nm. A high-resolution TEM (HRTEM) image (Figure 4d) demonstrates that nanoparticles are well crystallized. In the image, 2.0 Å lattice fringes corresponding to (200) planes of Au crystallized in the cubic structure are easily observed.

### 3.3. XPS Characterization

**Figure 5:**
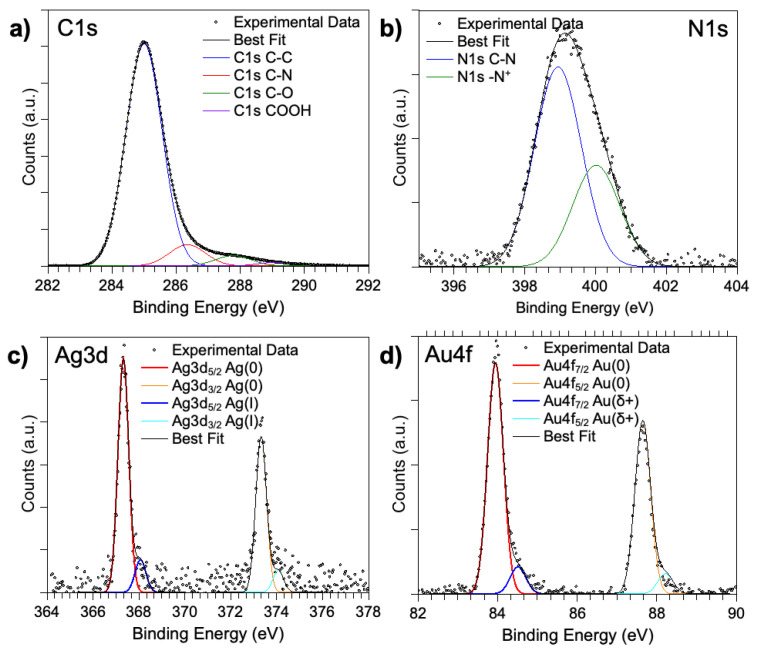
SR-XPS spectra collected at (**a**) C1s, (**b**) N1s, (**c**) Ag3d and (**d**) Au4f core levels on the pristine AuNRs.

Synchrotron radiation-induced X-ray photoelectron spectroscopy (SR-XPS) measurements were carried out on the pristine AuNRs, on the [Cu(PTA)_4_]BF_4_ coordination compound, and on the AuNRs/[Cu(PTA)_4_]BF_4_ (namely, AuNR-Cu(I)) complex system deposited onto TiO_2_/Si(111) wafer surfaces by following a drop-casting procedure. Data collected on [Cu(PTA)_4_]BF_4_ were already published and will be briefly summarized here, and they will be used as a reference for the analysis of the more complicated AuNR-Cu(I) adduct [34]. All SR-XPS data were analyzed by following a peak-fitting procedure, allowing us to identify the components arising from the chemical elements with different atomic environments; all peak-position BEs (binding energies), FWHM values, atomic ratios (relative intensities), and assignments are reported in Supporting Information Table S3. For AuNRs, C1s, N1s, Ag3d and Au4f core-level signals were collected and analyzed. The C1s spectrum is reported in Figure 5a. The spectrum appears to be a composite, and at least four spectral components can be individuated by following a peak-fitting procedure: the peak at the lowest BE (285.0 eV) is due to aliphatic C–C groups of ascorbate and impurities, always found on samples prepared in air; the peaks at about 286.4 and 287.8 eV are attributed to C–N and C–OH functional groups of ascorbate; the contribution at 289.2 eV is attributed to COOH impurities on the sample surface. The N1s spectrum (Figure 5b) is made of two components: the first at the lowest BE is associated with C–N nitrogens; the other, at around 400.0 eV, is associated with protonated nitrogen of CTAB. The Ag3d spectrum (Figure 5c) shows two pairs of spin–orbit (Ag3d_5/2_, Ag3d_3/2_) doublets, of which we consider the Ag3d_5/2_ component as the reference. The Ag3d_5/2_ signal at 367.3 eV is due to metallic bulk Ag(0) atoms, while those around 368.1 eV are due to substrate interface Ag atoms of the nanorods. The Au4f spectrum (Figure 5d) shows two pairs of spin–orbit (Au4f_7/2_ and Au4f_5/2_) doublets, of which we consider the Au4f_7/2_ component as the reference. The Au4f_7/2_ signal around 83.9 eV is due to metallic bulk Au(0) atoms of NRs, while the one at 84.5 eV arises from surface NR Au atoms.

**Figure 6:**
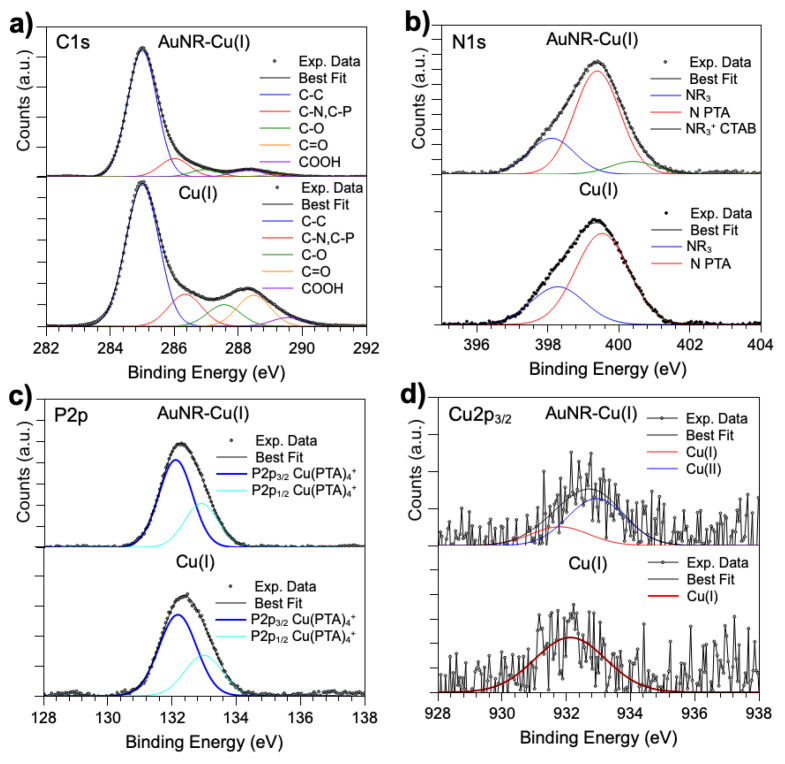
High-resolution SR-XPS spectra peak fit results for AuNR-Cu(I), compared with the same core level region measured on a pristine Cu(I) complex: (**a**) C1s; (**b**) N1s; (**c**) P2p; (**d**) Cu2p3/2.

SR-XPS data collected on AuNR-Cu(I) were analyzed and compared with [Cu(PTA)_4_]BF_4_ (namely, the Cu(I) complex) and with the data collected on AuNRs and discussed above. The C1s spectrum of AuNR-Cu(I) (Figure 6a, top) shows all the components already observed in the pristine AuNR and Cu(I) complex, as expected: the C-C signal is at 285.0 eV BE, C-N is superimposed with a C-P contribution at about 286 eV BE, C-O is at 287 eV, C=O is around 288 eV BE, and a very low peak arising from COOH groups at a high BE (289 eV). It is noteworthy that the last three contributions in Cu(I) arise from adventitious carbon impurities, as already observed in other works [33]. As for N1s and P2p signals (Figure 6b,c), they both indicate the successful functionalization of AuNRs with a Cu(I) complex. In fact, the N1s spectrum of AuNR-Cu(I) contains the two main features expected for the pristine Cu(I) coordination compound, as well as a third component at high BE values (401 eV) due to the positively charged quaternary amine of CTAB, while the P2p spectra observed in [Cu(PTA)_4_]BF_4_ and AuNR-Cu(I) are analogous, also confirming the chemical stability of the Cu(I) complex upon interaction with the AuNR surface. Finally, the Cu2p_3/2_ spectra collected at high resolution on the pristine Cu(I) complex and AuNR-Cu(I) show that the copper ion is partially oxidized from Cu(I) to Cu(II) in the nanosystem (Figure 6d). However, this is an expected behavior since the conjugation procedure is carried out in an aqueous environment; indeed, this finding was already observed and discussed in a recent publication [33]. Furthermore, the prolonged exposure to the X-ray beams necessary to acquire good data on the very diluted AuNP-Cu(I) sample could induce copper oxidation at the surface; this is highly visible in the XPS spectrum since this is a surface-sensitive technique with a sampling depth of 3–5 nm [53].

### 3.4. NEXAFS Characterization

**Figure 7:**
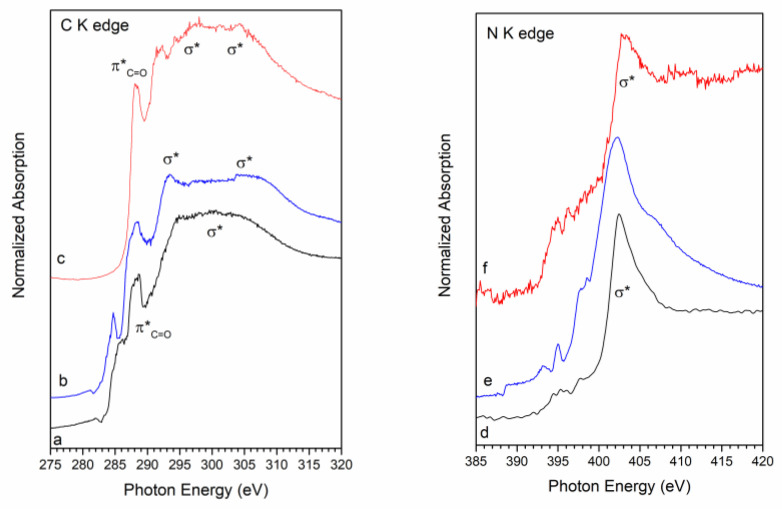
C K edge NEXAFS spectra of (a) AuNRs, (b) [Cu(PTA)_4_]BF_4_, (c) AuNR-Cu(I); N K edge NEXAFS spectra of (d) AuNRs, (e) [Cu(PTA)_4_]BF_4_, (f) AuNR-Cu(I).

NEXAFS spectroscopy measurements were carried out at the C and N K-edges on all samples in a solid state, prepared as samples for SR-XPS. The NEXAFS C K-edge spectrum of AuNR is reported in Figure 7a. The feature at about 288.7 eV is associated with the C 1s → π* transition of the C=O molecular orbital due to residual ascorbic acid molecules adsorbed on the nanorods surface; a shoulder at about 288 eV with a σ* resonance caused by the C−H groups and Rydberg features; and additional features around 293 and 303 eV, which can be assigned to 1s → σ* transitions by C−C and C=O molecular groups, respectively [54,55]. The peak at 288.7 eV also appears in the spectrum of [Cu(PTA)_4_]BF_4_ (Figure 7b), while the σ* resonance at 293 eV appears to be much more intense due to contributions from the many C-C bonds of the PTA ligand. The C K edge spectrum of the AuNR-Cu(I) sample (Figure 7c) appears as a combination of the spectra of [Cu(PTA)_4_]BF_4_ and AuNRs. The N K-edge spectrum of AuNR (reported in Figure 7d) shows a sharp peak at 402 eV, assigned to the N 1s → σ* transition of the positively charged nitrogen of CTAB. In the spectrum of [Cu(PTA)_4_]BF_4_ (Figure 7e), this peak appears to be more intense, with a shoulder at about 405 eV; pre-edge transitions, possibly due to Rydberg features, are also evident below the edge. Again, the C K spectrum of the AuNR-Cu(I) sample (Figure 7f) is a combination of the spectra of [Cu(PTA)_4_]BF_4_ and AuNRs.

### 3.5. FTIR Characterization

**Figure 8:**
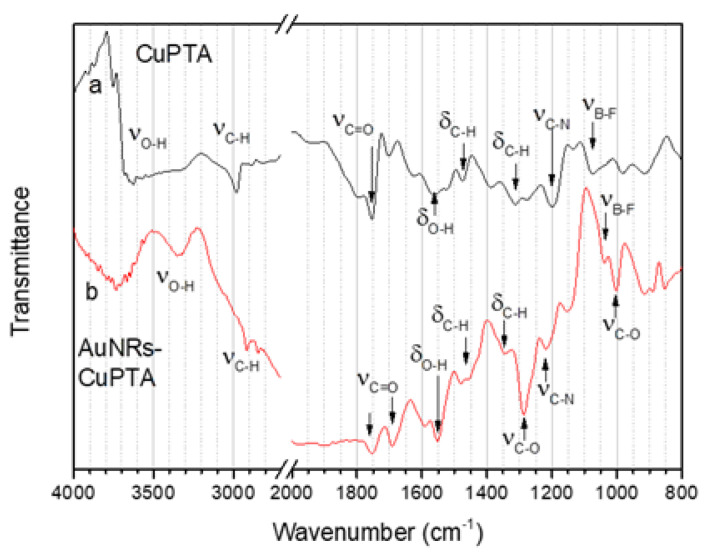
FTIR-spectra of (a) [Cu(PTA)_4_]BF_4_ and (b) AuNR-Cu(I) in the 4000–2700 cm^−1^ and 2000–800 cm^−1^ regions.

The FTIR spectra of the [Cu(PTA)_4_]BF_4_ complex and of the AuNR-Cu(I) conjugate in the spectral regions where the most diagnostic peaks are located are shown in Figure 8. The high wavenumber region (4000–2700 cm^−1^) of the spectrum of [Cu(PTA)_4_]BF_4_ (Figure 8a) shows peaks related to the stretching vibrations of C-H (ν_C-H_ 2890 cm^−1^) and O-H (ν_O-H_ at about 3500–3600 cm^−1^); the latter is due to physisorbed water. The bending vibrations of the corresponding bonds are found at 1470 and 1330 cm^−1^ (δ_C-H_) and at 1560 cm^−1^ (δ_O-H_). The C-N stretching vibration due to the PTA ligand (ν_C-N_) is located at 1200 cm^−1^, while the peak at 1070 cm^−1^ (ν_B-F_) can be assigned to the BF_4_ anion. The spectrum also presents a C=O stretching band at about 1750 cm^−1^ (ν_C=O_) due to residual ascorbic acid molecules adsorbed on the surface of the nanorods. The spectrum of the AuNR-Cu(I) conjugate is dominated by vibrations related to the ascorbic acid adsorbed on the AuNRs surface. Besides the peaks related to C-H stretching (ν_C-H_) and bending (δ_C-H_), which are found approximately in the same positions as the corresponding peaks in the spectrum of the Cu(I) complex, we observe an intense O-H stretching (ν_O-H_) located at 3350 cm^−1^ and related to the many O-H groups of ascorbic acid. Two peaks related to C=O stretching bands are found at 1750 and 1680 cm^−1^; the second one is due to the ester moiety of the ascorbic acid. Peaks related to the stretching of the C-O bonds of the ester and alcohol functions of ascorbic acid are found at 1280 and 1020 cm^−1^ respectively. The C-N stretching band of [Cu(PTA)_4_]BF_4_ (νC-N) and the band due to the BF_4_^−^ anion (ν_B-F_) are also visible in the spectrum as a consequence of the Cu(I) complex immobilization on the AuNR surface [56].

### 3.6. Biological Tests

**Figure 9:**
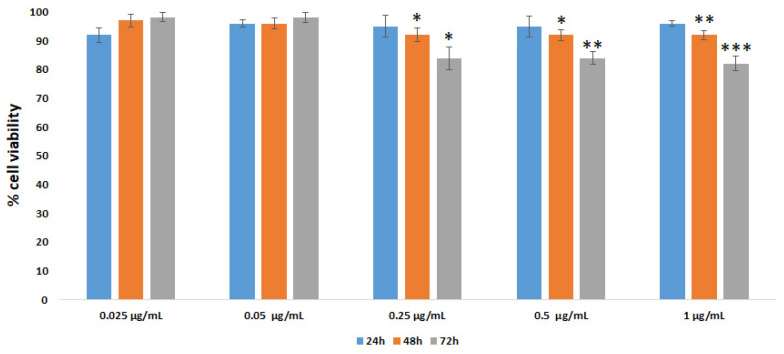
Vero E6 cell viability evaluated by the MTT test. Cells were incubated with AuNRs at different concentrations and treatment times. Data are expressed as % of cell viability with respect to the control (set at 100% value). All experiments were conducted in triplicate in at least three independent experiments. Results are expressed as mean ± SD values. * *p* < 0.05, ** *p* < 0.01, *** *p* < 0.001 (GraphPad Prism 10, unpaired *t*-test).

To analyze the biocompatibility of AuNRs, the Vero E6 cell line was employed. This normal/immortalized cell line represents a useful model to perform viral propagation, vaccine production and biocompatibility assays. Vero E6 cells were treated with different concentrations of AuNRs (0.025, 0.05, 0.25, 0.5 and 1 µg/mL) for 24, 48, and 72 h, and the measurement of cell viability was carried out by an MTT assay. The results are shown in Figure 9 and are in agreement with other studies performed with biocompatible AuNRs [57,58,59,60].

**Figure 10:**
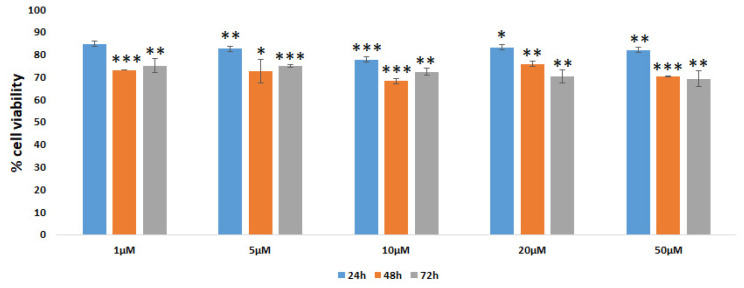
Vero E6 cell viability evaluated by MTT test. Cells were incubated with [Cu(PTA)_4_]BF_4_ at different concentrations and treatment times. Data are expressed as % of cell viability with respect to the control (set at 100% value). All experiments were conducted in triplicate in at least three independent experiments. Results are expressed as mean ± SD values. * *p* < 0.05, ** *p* < 0.01, *** *p* < 0.001 (GraphPad Prism 10, unpaired *t*-test).

Next, Vero cells were incubated with the [Cu(PTA)_4_]BF_4_ coordination compound employed at concentrations between 1 and 50 µM for 24, 48 and 72 h to analyze the biocompatibility. It can be seen that this coordination compound induced a small reduction in cell viability (nearly 15–20%) after the 24 h treatment, which reached approximately 30% after the 48 and 72 h treatments (Figure 10). This behavior is consistent with analogous studies [61,62].

**Figure 11:**
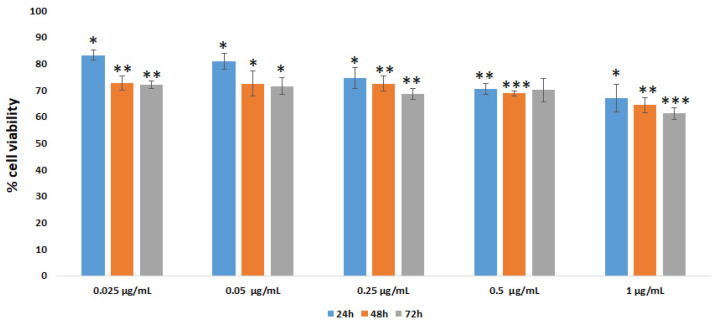
Vero E6 cell viability evaluated by MTT test. Cells were incubated with AuNR-Cu(I) at different concentrations and treatment times. Data are expressed as % of cell viability with respect to the control (set at 100% value). All experiments were conducted in triplicate in at least three independent experiments. Results are expressed as mean ± SD values. * *p* < 0.05, ** *p* < 0.01, *** *p* < 0.001 (GraphPad Prism 10, unpaired *t*-test).

Finally, the effect of treatment with different concentrations of AuNR-Cu(I) complex (obtained after conjugation of AuNRs with the [Cu(PTA)_4_]BF_4_ coordination compound) on Vero E6 cell viability was evaluated by an MTT assay for up to 72 h. As shown in Figure 11, a slight reduction in cell viability could be observed after treatments with 0.025 and 0.05 µg/mL for 24 h (a 20% reduction value as calculated with respect to a control sample set at 100%). Incubation for longer times or with higher AuNR-Cu(I) conjugate doses (0.25, 0.5 and 1 µg/mL) caused a minimal cell viability reduction (30 to 40%).

The results obtained from the biological assessments indicated that the AuNRs, the Cu coordination compound [Cu(PTA)_4_]BF_4_, and the AuNR-Cu(I) conjugate induced no or a very light reduction in Vero E6 cell viability, strongly suggesting good biocompatibility. These results are a promising example of the use of our selected copper complexes conjugated with AuNRs as versatile biocompatible anisotropic chemical drug delivery systems. In fact, this conjugate system will apply to applications in antiviral treatment strategies, using Vero cells as a reference normal culture or cell-culture-based infection model for viral replication [63].

## 4. Conclusions

In this work, the synthesis of highly hydrophilic gold nanorods was studied and implemented, exploiting the two-step wet method. The objective of obtaining a system for the transport of copper(I)-based drugs was thus achieved. TEM observations showed AuNRs with regular shapes and sizes, with an AR of 2.6, confirmed by DLS and ζ potential measurements. The AuNRs were conjugated with a Cu(I)-based drug, i.e., [Cu(PTA)_4_]BF_4_ (PTA = 1,3,5- triaza-7-phosphadamantane), obtaining a loading efficiency of η (%) = 50 ± 10%. The conjugate system was characterized using different spectroscopies (UV–Vis–NIR, FT-IR, and XPS), which verified the surface functionalization and Cu(I)-Au interaction. To study the biocompatibility of conjugated and non-conjugated AuNRs, MTT assays were performed with Vero E6 cells. These experiments showed a reduction in biocompatibility only for high concentrations (1 µg/mL) and long times (72 h), confirming the high applicative potential of the conjugated system in nanomedicine and, in particular, for innovative antiviral treatment strategies.

## Data Availability

The original contributions presented in this study are included in the article/Supplementary Materials. Further inquiries can be directed to the corresponding author.

## Supplementary Materials

The following supporting information can be downloaded at https://www.mdpi.com/article/10.3390/nano16030217/s1: Table S1: Experimental details of gold nanorod synthesis. Table S2: Experimental details of drug conjugation. Figure S1: (a) ζ potential measurements of AuNRs performed immediately after synthesis (blue line, ζ potential = 46 ± 4 mV) and after 3 months (orange line, ζ potential = 53 ± 6 mV); (b) ζ potential measurements of AuNR-Cu(I) performed after 9 months from conjugation, ζ potential = −27 ± 7 mV. Table S3: XPS data (BE, FWHM, atomic ratio values and proposed assignments) collected from AuNRs, AuNR-Cu(I) and Cu(I) complex.
