# Supplementary material for: Conjugation of Functionalized Gold Nanorods and Copper (I)-Based Drug: An Anisotropic Nano Drug Delivery System
Source: Nanomaterials (Basel). 2026 Feb 6;16(3):217. doi: 10.3390/nano16030217 (PMC12899677; doi:10.3390/nano16030217)
Supplement: Supplementary file 1 [file nanomaterials-16-00217-s001.zip › nanomaterials-4108334-supplementary.pdf]

## SUPPLEMETARY MATERIAL

**SM-Table S1.** Experimental details of gold nanorods synthesis.

| Reaction conditions | | |
|---|---|---|
| | V | M |
| CTAB | 10 ml | 0.2 M |
| $HAuCl_4$ | 10 ml | 0.001M |
| Ascorbic Acid | 140 µl | 0.078 M |
| seed solution | v.p. * | |
| $AgNO_3$ | 400 µl | v.p.** |

| Reaction conditions v.p.* | | |
|---|---|---|
| | V | M |
| CTAB | 10 ml | 0.2 M |
| $HAuCl_4$ | 10 ml | 0.001M |
| Ascorbic Acid | 140 µl | 0.078 M |
| seed solution | 24 µL | |
| $AgNO_3$ | 400 µl | 0.004 M |

| Reaction conditions v.p.* | | |
|---|---|---|
| | V | M |
| CTAB | 10 ml | 0.2 M |
| $HAuCl_4$ | 10 ml | 0.001M |
| Ascorbic Acid | 140 µl | 0.078 M |
| seed solution | 12 µL | |
| $AgNO_3$ | 400 µl | 0.004 M |

| Reaction conditions v.p.** | | |
|---|---|---|
| | V | M |
| CTAB | 10 ml | 0.2 M |
| $HAuCl_4$ | 10 ml | 0.001M |
| Ascorbic Acid | 140 µl | 0.078 M |
| seed solution | 24 µL | |
| $AgNO_3$ | 400 µl | 0.002 M |

| Reaction conditions v.p.** | | |
|---|---|---|
| | V | M |
| CTAB | 10 mL | 0.2 M |
| $HAuCl_4$ | 10 mL | 0.001M |
| Ascorbic Acid | 140 µL | 0.078 M |
| seed solution | 24 µL | |
| $AgNO_3$ | 400 µL | 0.008 M |

**SM-Table S2.** Experimental details of drug conjugation.

| **Drug Conjugation** | |
|---|---|
| AuNRs (mg/mL) | 0.6 |
| Cu (I) (mg/mL) | 0.1 |
| Time (h) | 24 |
| Temperaaure ( °C) | 25 |
| | |

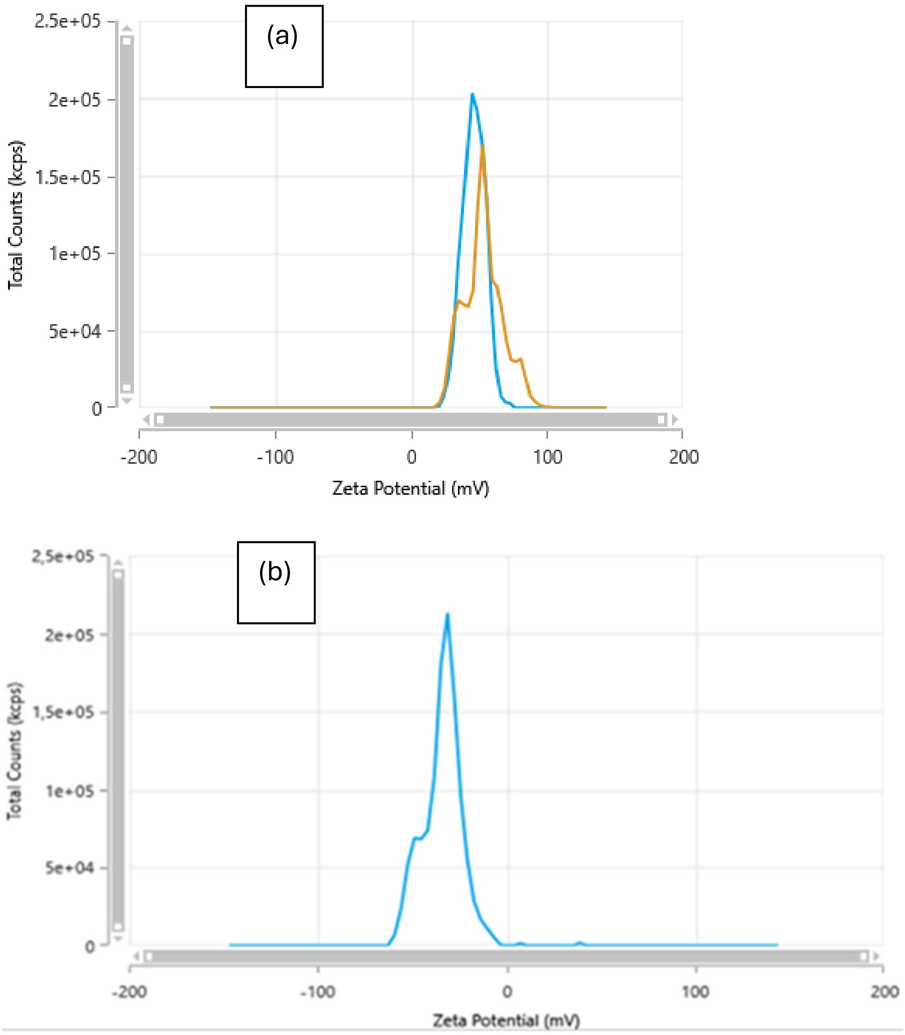


**SM-Figure S1.** (a) ζ potential measurements of AuNRs performed immediately after synthesis (blue line, ζ potential = 46 ± 4 mV) and after 3 months (orange line ζ potential = 53 ± 6 mV); (b) ζ potential measurements of AuNRs-Cu(I) performed after 9 month from conjugation, ζ potential = -27 ± 7 mV.

| **SM-Table S3.** XPS data (BE, FWHM, Atomic Ratio values and proposed assignments) collected on AuNRs, AuNRs-Cu(I) and Cu(I) complex. | | | | | |
|---|---|---|---|---|---|
| **Sample** | **Signal** | **BE (eV)** | **FWHM** | **Atomic Ratios (%)** | **Assignments** |
| AuNRs | C1s | 285.00<br>286.35<br>287.78<br>289.15 | 1.34 | 86<br>8<br>4<br>2 | C-C<br>C-N<br>C-O<br>COOH |
| | N1s | 398.94<br>400.02 | 1.57 | 66<br>34 | C-N<br>$-N^{+}$ |
| | Au4f | 83.96<br>84.54 | 0.47 | 90<br>10 | $Au^{0}$<br>$Au^{\delta+}$ |
| | Ag3d | 367.32<br>368.07 | 0.56 | 88<br>12 | $Ag^{0}$<br>$Ag^{\delta+}$ |
| AuNRs-Cu(I) | C1s | 285.00<br>286.02<br>286.96<br>288.28<br>289.19 | 1.10 | 78<br>12<br>4<br>4<br>2 | C-C<br>C-N,C-P<br>C-O<br>C=O<br>COOH |
| | N1s | 398.11<br>399.39<br>400.41 | 1.50 | 27<br>68<br>5 | <br>C-N<br>$-N^{+}$ |
| | Au4f | 83.96<br>84.39 | 0.47<br>1.17 | 85<br>15 | $Au^{0}$<br>$Au^{\delta+}$ |
| | Ag3d | 366.62<br>367.28 | 0.58 | 76<br>24 | $Ag^{0}$<br>$Ag^{\delta+}$ |
| | O1s | 530.81<br>532.14<br>533.43 | 1.50 | 59<br>36<br>5 | C=O<br>C-O<br>Physisorbed $H_2O$ |
| | $P2p_{3/2}$ | 132.10 | 1.23 | 100 | $Cu(PTA)_4^{+}$ |
| | $Cu2p_{3/2}$ | 931.87<br>932.98 | 2.12 | 30<br>70 | Cu(I) $Cu(PTA)_4^{+}$<br>Cu(II) |
| Cu(I) | C1s | 285.00<br>286.35<br>287.56<br>288.48<br>289.56 | 1.22 | 60<br>14<br>9<br>13<br>4 | C-C<br>C-N,C-P<br>C-O<br>C=O<br>COOH |
| | N1s | 398.28<br>399.53 | 1.75 | 30<br>70 | C-N<br>$-N^{+}$ |
| | O1s | 530.97<br>532.39 | 1.78 | 76<br>24 | C=O<br>C-O |
| | $P2p_{3/2}$ | 132.19 | 1.37 | 100 | $Cu(PTA)_4^{+}$ |
| | $Cu2p_{3/2}$ | 932.08 | 2.06 | 100 | Cu(I) $Cu(PTA)_4^{+}$ |
